# GMEB2 Promotes the Growth of Colorectal Cancer by Activating ADRM1 Transcription and NF-κB Signalling and Is Positively Regulated by the m^6^A Reader YTHDF1

**DOI:** 10.3390/cancers14246046

**Published:** 2022-12-08

**Authors:** Zhengping Ning, Zhiwei Wu, Fan Zhang, Ming Yang, Zhixing Lu, Bowen Yu, Fei Long, Yihang Guo, Kaiyan Yang, Gui Hu, Yi Zhang, Xiaorong Li, Liang Li, Changwei Lin

**Affiliations:** 1 Department of Gastrointestinal Surgery, The Third XiangYa Hospital of Central South University, Changsha 410013, China; 2 Department of Gastrointestinal, Hernia and Enterofistula Surgery, People’s Hospital of Guangxi Zhuang Autonomous Region, Nanning 530022, China

**Keywords:** colorectal cancer, GMEB2, RNA N6-methyladenosine, YTHDF1, ADRM1, NF-κB

## Abstract

**Simple Summary:**

Colorectal cancer (CRC) is the third most common cancer worldwide and the second leading cause of cancer-related death. In this study, we aimed to determine the biological function and regulatory mechanism of GMEB2 in CRC. We found that GMEB2 was highly expressed in CRC and significantly promoted the growth of CRC in vitro and in vivo. Mechanistically, GMEB2 acted as a transcription factor to activate ADRM1/NF-κB signalling and was upregulated by YTHDF1 through enhancing its mRNA stability. Our findings suggest that GMEB2 may serve as a new therapeutic target for CRC treatment in the future.

**Abstract:**

Transcription factors are frequently aberrantly reactivated in various cancers, including colorectal cancer (CRC). However, as a transcription factor, the role of GMEB2 in cancer is still unclear, and further studies are needed. Here, we aimed to identify the function and mechanism of GMEB2 in regulating the malignant progression of CRC. GMEB2 was found to be highly expressed in online data analyses. We demonstrated that GMEB2 was markedly upregulated at both the mRNA and protein levels in CRC cells and tissues. GMEB2 knockdown inhibited CRC cell growth in vitro and in vivo. Mechanistically, as a transcription factor, GMEB2 transactivated the ADRM1 promoter to increase its transcription. Rescue experiments showed that ADRM1 downregulation partially reversed the promoting effects of GMEB2 on CRC growth in vitro. Moreover, the GMEB2/ADRM1 axis induced nuclear translocation of NF-κB, thus activating NF-κB signalling. Finally, we further revealed that YTHDF1 recognized and bound to the m^6^A site on GMEB2 mRNA, which enhanced its stability. Taken together, our findings reveal the crucial role and regulatory mechanism of GMEB2 in CRC for the first time and provide a novel potential therapeutic target for CRC therapy.

## 1. Introduction

Colorectal cancer (CRC) is one of the most common malignancies worldwide, with the third highest morbidity and second highest mortality [1]. Colorectal cancer is expected to cause 52,580 deaths in the United States in 2022 [2]. The incidence of colorectal cancer in China is still rising, with an estimated 5,932,232 new CRC patients in 2022 [3]. Although the treatment of colon cancer has improved with the enhancement of medical services, due to a lack of diagnostic biomarkers and therapeutic targets, the five-year survival rate of CRC is only 64% [4]. Therefore, it is necessary to further explore the pathogenesis of colorectal cancer and new directions of treatment.

Glucocorticoid modulatory element-binding proteins (GMEBs), including GMEB1 and GMEB2, are members of the KDWK protein family, and each displays intrinsic transactivation activity as a transcription factor [5]. GMEBs share a similar DNA-binding domain, called the SAND domain, for transcriptional activities with 80% identity, and 39% of GMEBs are homologous [6]. Moreover, GMEB2 has more potent transactivation properties than GMEB1 [6] and is more highly expressed in certain human tissues, such as the colon and liver [7]. Even though GMEBs were found to regulate the transactivation of glucocorticoid receptors [8], their respective target genes and signalling pathways for which they act as transcription factors remain unknown. Some studies have reported that GMEB1 contributes to tumour development [9,10,11] and inhibits cell apoptosis [12,13,14]. However, there is no study that has specifically focused on the transcription factor GMEB2. The biological function and regulatory mechanism of GMEB2 in cancer are still not clear.

Interestingly, a recent study found a N6-methyladenosine (m^6^A) modification on GMEB2 mRNA, which may be recognized by the m^6^A reader YTHDF1 in tumour cells [15]. M^6^A is the most extensive internal epitranscriptomic modification of mRNA in eukaryotes [16,17], influencing the entire life cycle of mRNAs, including their stability and translation [18,19,20]. Disorders of m^6^A writers (methyltransferases), erasers (demethylases) or readers (m^6^A-binding proteins) lead to abnormal m^6^A modification of mRNAs, which impacts the expression of many genes in cancer [21,22]. Among them, YTHDF1 is the most versatile and powerful m^6^A reader protein and is involved in the regulation of diverse cancers. It is believed that YTHDF1 promotes mRNA translation by recognizing m^6^A sites to regulate cancer progression [23]. However, a few studies have recently reported that YTHDF1 can also enhance the stability of mRNAs, such as PNN [24], by recognizing METTL3-mediated m^6^A modification. Therefore, whether GMEB2 mRNA is regulated by YTHDF1 in an m^6^A-dependent manner in cancer deserves further investigation.

Adhesion Regulating Molecule-1 (ADRM1) protein is a component of the 19S regulatory particle subcomplex in the 26S proteasome, which recognizes K48-linked polyubiquitinated proteins and promotes their degradation [25]. Recent studies have shown that ADRM1 is overexpressed [26] and promotes the proliferation and metastasis of CRC [27,28,29], liver cancer [30], gastric cancer [31,32,33] and ovarian cancer [34,35]. Moreover, ADRM1 is a therapeutic target for cancer treatment. Dysfunction of ADRM1 restricts NF-κB translocation from the cytosol to the nucleus and thus blocks NF-κB signalling [36,37], which induces tumour cell apoptosis [38]. However, the upstream regulatory mechanism of ADRM1 has not been elucidated. Transcription factors are frequently aberrantly reactivated in cancer, stimulating the transcription of various oncogenes. Whether the overexpression of ADRM1 is regulated by transcription factors in cancer requires more research.

Here, we evaluated GMEB2 expression in CRC and then explored the transcriptional activation function and regulatory mechanisms of GMEB2 in CRC. The aim of this study was to identify the important role of GMEB2 in CRC development and provide a potential therapeutic target for CRC treatment.

## 2. Materials and Methods

### 2.1. Samples and Databases

Clinical samples were collected during surgery from the Third Xiangya Hospital of Central South University (Changsha, China) with informed consent and approval of the Medical Ethics Central South University (No:22199). Tissue specimens were snap-frozen and stored in liquid nitrogen before detection. The expression data of GMEB2, ADRM1 and YTHDF1 were downloaded from the TCGA (https://gdc.cancer.gov/, accessed on 10 March 2021) and GEO (https://www.ncbi.nlm.nih.gov/geo, accessed on 10 March 2021) databases. The correlated genes of GMEB2 were analyzed using the GEPIA (http://gepia.cancer-pku.cn/, accessed on 10 March 2021) database.

### 2.2. Cell Lines and Cell Culture

Colorectal cancer cell lines were purchased from KeyGEN BioTECH (Jiangsu, China). FHC was purchased from American Type Culture Collection (ATCC) (Manassas, VA, USA). All cell lines were free of mycoplasma contamination and were authenticated using STR (or SNP) profiling within the last 3 years. SW480 cells were cultured with L15 (KeyGEN BioTECH, Jiangsu, China) medium. HT29 and HCT116 cells were cultured with McCoy’s 5 A (KeyGEN BioTECH) medium. FHC cells were cultured with Roswell Park Memorial Institute 1640 (RPMI 1640; KeyGEN BioTECH) medium. All culture media were supplemented with 10% fetal bovine serum (FBS; Biological Industries, Israel) and 1% antibiotics (100 U/mL of penicillin and 100 mg/mL of streptomycin; Life Technologies, Inc., Grand Island, NY, USA). All cells were grown at 37 °C with 5% CO_2_.

### 2.3. RNA Extraction and Quantitative Real-Time PCR (qRT-PCR) Analysis

Total RNA from cells and tissues was extracted using TRIzol Reagent (Invitrogen, Carlsbad, CA, USA). Complementary DNA was synthesized using the HiScript III RT SuperMix (Vazyme, Nanjing, China). Quantitative real-time PCR (qRT-PCR) was performed using a LightCycler 480 Real Time PCR instrument (Roche, Basel, Switzerland). GAPDH was used to normalize the qRT-PCR data. All primer sequences are listed in Supplementary Table S1.

### 2.4. Western Blotting

Cells were lysed in 1× RIPA buffer (KeyGEN BioTECH) containing 1% PMSF (KeyGEN BioTECH) to harvest proteins. The proteins were separated by 10% sodium dodecyl sulfate-polyacrylamide gel electrophoresis (SDS-PAGE) and transferred to polyvinylidene fluoride membranes (PVDF) (Millipore, CA, USA). Nonspecific binding was blocked with 5% non-fat milk at room temperature for 1 h. Membranes were then incubated with primary antibodies at 4 °C overnight and secondary antibodies for 1 h. Finally, the proteins were visualized using an Odyssey CLx Infrared Imaging System (LI-COR Biosciences, NE, USA). The antibodies in this study are provided in Supplementary Table S2.

### 2.5. Immunohistochemistry (IHC) Staining

Tumor tissues obtained from CRC patients or xenografts were embedded in paraffin and then cut into 4 μm sections. Briefly, sections were deparaffinized and boiled in Tris antigen-retrieval buffer. Then, sections were incubated with primary antibodies against GMEB2 (Bioss, 1:200 dilution) overnight at 4 °C. Subsequently, secondary antibodies were incubated for 1 h at 37 °C. Finally, the samples were stained and imaged.

### 2.6. Plasmid Construction, Cell Transfection and Lentivirus Vector Infection

All overexpression plasmids (YTHDF1, GMEB2, ADRM1), shRNA plasmids (sh-METTL3, sh-YTHDF1, sh-GMEB2, sh-ADRM1) and negative control plasmids were constructed by Tsingke Biotechnology Co., Ltd. Their corresponding lentivirus productions were also provided. To generate stable knockdown or overexpression cell lines, cells were infected with lentivirus using HitransG P (Genechem, Shanghai, China) in accordance with the instructions and were selected with 4 µg/mL of puromycin after 48 h of infection. For transient transfection, the cells were seeded into six-well plates and cultured for 24 h. When the density reached 50–60%, Lipofectamine 3000 reagent (Invitrogen, Carlsbad, CA, USA) was used to transfect the plasmids according to the manufacturer’s instructions. Related interference sequences are listed in Supplementary Table S1.

### 2.7. Cell Proliferation and Colony Formation Assay

The cell counting kit (CCK-8) assay and 5-ethynyl-2′-deoxyuridine (EdU) proliferation assay were used to measure cell proliferation. Approximately 4000 HCT116, HT29 and SW480 cells/well were seeded into 96-well plates. CCK8 (Dojindo, Kumamoto, Japan) was added at 0, 24, 48 and 72 h, followed by incubation at 37 °C for 2 h. The absorbance at 450 nm was detected using an EnVision microplate reader (PerkinElmer). The EdU Cell Proliferation Assay Kit (KeyGEN, Nanjing, China) was used according to the manufacturer’s instructions to perform the EdU assay. For colony formation assay, approximately 500 treated cells/well were seeded into 6-well plates in replicates of three. After a 14-day incubation, these colonies were fixed with methanol and stained with 0.1% crystal violet for further counting.

### 2.8. Tumor Xenografts

The xenograft experiment was performed according to the guidelines for experimental animal management established by Kagawa University and guidelines for the welfare and use of animals in cancer research [39]. Male BALB/c nude mice (4–5 weeks, 18–20 g) were obtained from the Department of Laboratory Animals of Central South University and maintained under specific pathogen-free conditions. HT29 and SW480 cells (2 × 10^6^) transfected with GMEB2 shRNA or negative control RNA were injected subcutaneously into the left or right flank of the nude mice (*n* = 5 per group). Tumor size was estimated using calipers and calculated based on the formula: volume (mm^3^) = length (mm) × width (mm) × width (mm)/2. Thirty-five days after the injection, the mice were sacrificed, and the final tumor volume and weight data were recorded.

### 2.9. Chromatin Immunoprecipitation (ChIP) Assay

Chromatin immunoprecipitation (ChIP) assay was performed using the ab500 ChIP Kit (Abcam, Cambs, UK) according to the manufacturer’s instructions. Briefly, SW480 cells were sonicated to yield optimal DNA fragment size of 200–1000 bp. Immunoprecipitation (IP) was carried out using the mouse monoclonal GMEB2 antibody (Santa Cruz) at 4 °C overnight. An unrelated mouse IgG was used as a negative control. Finally, qRT-PCR was performed on purified input and immunoprecipitated DNA. The primer sequences for promoter region are listed in Supplemental Table S1.

### 2.10. Dual-Luciferase Reporter Assay

Wild-type, truncation and site-directed mutagenesis of ADRM1 promoter were, respectively, inserted into the GV238 vector (Genechem, Shanghai, China). Then, 293 T cells were seeded on 24-well plates and co-transfected with plasmids carrying GMEB2, Renilla luciferase and ADRM1 promoter using Lipofectamine 3000 (Invitrogen, CA, USA). After 48 h, luciferase activity was measured by the Dual-Luciferase Reporter Assay System (Promega, WI, USA).

### 2.11. Immunofluorescent Staining

Cells were incubated with the primary antibody against NF-κB p65 (Zenbio, 1:200 dilution) overnight at 4 °C. Secondary antibodies conjugated with Dylight Fluor were incubated for 1 h at 37 °C. Finally, the nucleus was stained with DAPI. The immunofluorescence signal was visualized by confocal microscopy using a Leica TCS SP8.

### 2.12. Methylated RNA Immunoprecipitation (MeRIP)

After extracting the total RNA (20 μg) of SW480 cells, the m^6^A RNA Enrichment Kit (EpiGentek, NY, USA) was used to enrich and purify the methylated RNA according to the manufacturer’s instructions. Finally, RNA reverse transcription PCR and qRT-PCR were performed to detect the m^6^A sites. The primer sequences for the m^6^A site are listed in Supplemental Table S1.

### 2.13. RNA Immunoprecipitation (RIP) Assay

An RNA immunoprecipitation assay was performed using the EZ-Magna RIP Kit (Merck, Darmstadt, Germany) according to the manufacturer’s instructions. In brief, SW480 cells were lysed in RIP lysis buffer. Then, the lysates were incubated with YTHDF1 polyclonal antibody (Proteintech, Chicago, IL, USA) overnight at 4 °C. Finally, RNA was extracted and purified for qRT-PCR analysis.

### 2.14. Statistical Analysis

Statistical analysis was performed using GraphPad Prism 8.0. (San Diego, CA, USA). The data are presented as means ± S.D. of three independent experiments. To assess the differences between two groups, Student’s *t*-test was performed. Two-way analysis of variance (ANOVA) was used for comparisons between the different groups. A value of *p* < 0.05 was considered statistically significant.

## 3. Results

### 3.1. GMEB2 Expression Is Elevated in CRC

**Figure 1:**
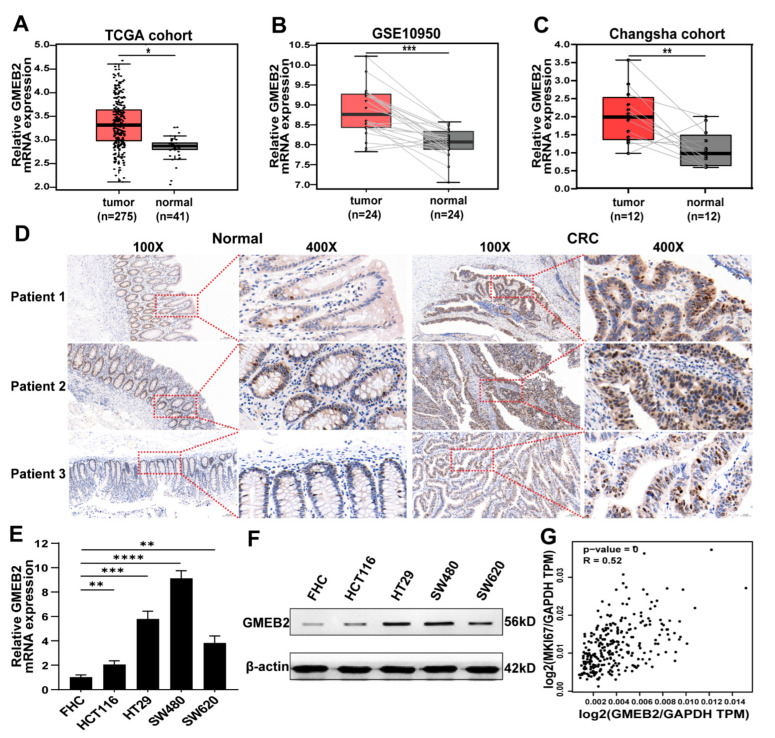
The mRNA and protein expression levels of GMEB2 in CRC. (**A**) GMEB2 mRNA level in the TCGA database (Student’s *t*-test, * *p* < 0.05). (**B**) GMEB2 mRNA level in GSE10950 of the GEO database (paired Student’s *t*-test, *** *p* <0.001). (**C**) GMEB2 mRNA levels in 12 pairs of CRC and paracancerous tissues from patients (paired Student’s *t*-test, ** *p* < 0.01). (**D**) GMEB2 protein levels in 3 pairs of CRC and paracancerous tissues from patients measured by IHC. Scale bar, 20 µm and 100 µm. (**E**) GMEB2 mRNA levels in FHC and CRC cell lines (HCT116, HT29, SW480 and SW620) (Student’s *t*-test, ** *p* < 0.01, *** *p* < 0.001, **** *p* < 0.0001). (**F**) GMEB2 protein levels in FHC and CRC cell lines (HCT116, HT29, SW480 and SW620) measured by western blotting. (**G**) Correlation of mRNA expression between GMEB2 and MKI67 in CRC from GEPIA database.

To explore the role of GMEB2 in CRC development, the expression of GMEB2 was first analysed in online databases. We found that GMEB2 was highly expressed in CRC by analysing The Cancer Genome Atlas (TCGA) database (Figure 1A). Next, we assessed GMEB2 expression in the downloaded CRC gene expression dataset GSE10950, in which CRC and adjacent tissues were paired, from the Gene Expression Omnibus (GEO) database. A consistent trend was observed (Figure 1B). For further confirmation, we detected GMEB2 expression in 12 matched pairs of human CRC tissues and adjacent non-tumour tissues by qRT-PCR and 3 matched pairs of tissues by immunohistochemistry (IHC). GMEB2 was found to be significantly upregulated at both the mRNA and protein levels in human CRC tissues (Figure 1C,D). Moreover, the GMEB2 expression level was examined in human normal colon epithelium cell line (FHC) and four CRC cell lines (HCT116, HT29, SW480 and SW620). GMEB2 expression was markedly higher in the CRC cell lines than in the FHC (Figure 1E,F and Figure S1A). Then, we selected SW480 and HT29 cells, which have relatively high GMEB2 expression, for GMEB2 knockdown and HCT116 cells for GMEB2 overexpression. In addition, GMEB2 expression in CRC was obviously correlated with the proliferation marker MKI67 in the Gene Expression Profiling Interactive Analysis (GEPIA) database (Figure 1G). Collectively, these results indicate that GMEB2 expression was upregulated in CRC.

### 3.2. GMEB2 Drives CRC Cell Growth In Vitro and In Vivo

**Figure 2:**
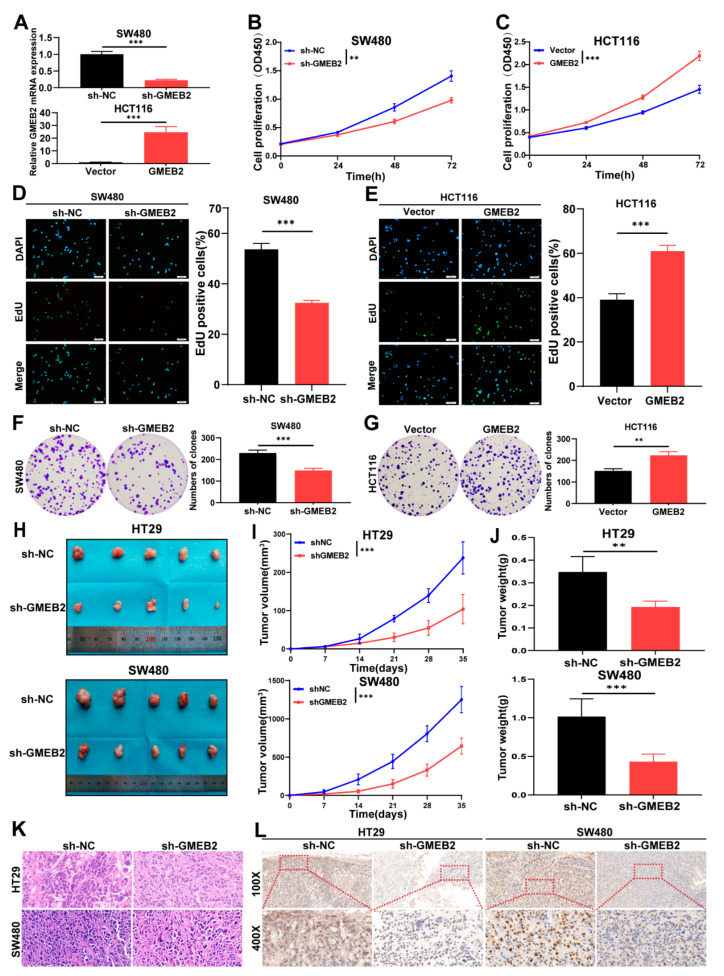
GMEB2 facilitates CRC cell growth in vitro and in vivo. (**A**) Silencing or overexpression efficiencies of GMEB2 in SW480 and HCT116 cells measured by qRT-PCR (Student’s *t*-test, *** *p* < 0.001). (**B**,**C**) CCK-8 assays were used to detect the proliferation of SW480 and HCT116 cells after knockdown or overexpressing GMEB2 (two-way ANOVA, ** *p* < 0.01, *** *p* < 0.001). (**D**,**E**) EdU assays and the relative quantitative results of cell proliferation are shown in histogram form (Student’s *t*-test, *** *p* < 0.001). (**F**,**G**) The colony formation assays and the numbers of clones were counted (Student’s *t*-test, ** *p* < 0.01, *** *p* < 0.001). The results are presented as the mean ± S.D. of three independent experiments. *p* < 0.05. (**H**) Images of xenograft-transplanted nude mouse models (*n* = 5) and dissected tumours 35 days after injection with shGMEB2 HT29 and SW480 cells and their corresponding NC cells. (**I**) Xenograft tumor growth curves of sh-GMEB2 cells and their corresponding NC cells (Student’s *t*-test, *** *p* < 0.001). (**J**) Xenograft tumor weight of sh-GMEB2 cells and their corresponding NC cells (Student’s *t*-test, ** *p* < 0.01, *** *p* < 0.001). (**K**) Representative images of H&E staining of mouse xenograft tumours. (**L**) GMEB2 IHC of mouse xenograft tumours. Scale bar, 20 µm and 100 µm.

Given that GMEB2 was highly correlated with the proliferation marker MKI67, we hypothesized that the expression of GMEB2 influences the proliferative function of CRC cells. To validate our hypothesis, we assessed the effects of GMEB2 knockdown or overexpression on CRC cell growth. We silenced GMEB2 in SW480 and HT29 cells with lentiviral vectors carrying GMEB2-specific small hairpin RNA (sh-GMEB2). Negative control cells were transfected with lentiviral vectors carrying scrambled shRNA (sh-NC). Meanwhile, we constructed HCT116 cells that stably overexpressed GMEB2 by transfecting lentiviral GMEB2 (GMEB2), and a lentiviral vector (Vector) was used as a control. The GMEB2 silencing and overexpression efficiencies were verified by qRT-PCR and western blotting (Figure 2A and Figure S1B,C). We demonstrated that the knockdown or overexpression of GMEB2 inhibited or promoted CRC cell proliferation activity, respectively, by CCK-8, EdU and colony formation assays (Figure 2B–G and Figure S1D–H). Then, we evaluated the effect of GMEB2 on cell apoptosis. The silencing of GMEB2 induced a significant increase in apoptotic cell death in SW480 and HT29 cells, as analysed by flow cytometry (Figure S1I). These data suggest that GMEB2 played an essential role in promoting CRC cell growth in vitro. To further confirm the role of GMEB2 in vivo, we injected stable GMEB2-knockdown HT29 and SW480 cells and their corresponding sh-NC cells into nude mice. Thirty-five days after implantation, GMEB2-silenced cells had formed much smaller tumours than sh-NC cells regarding tumour volume and weight (Figure 2H–J). The tumour tissues were sectioned and assessed by haematoxylin and eosin (H&E) staining (Figure 2K) and IHC (Figure 2L), which verified the lower GMEB2 expression in the GMEB2-knockdown tumour group than in the sh-NC group. Taken together, these results demonstrate that GMEB2 drove CRC cell growth in vitro and in vivo.

### 3.3. ADRM1 Is Transactivated by GMEB2, and Promotes CRC Cell Growth

**Figure 3:**
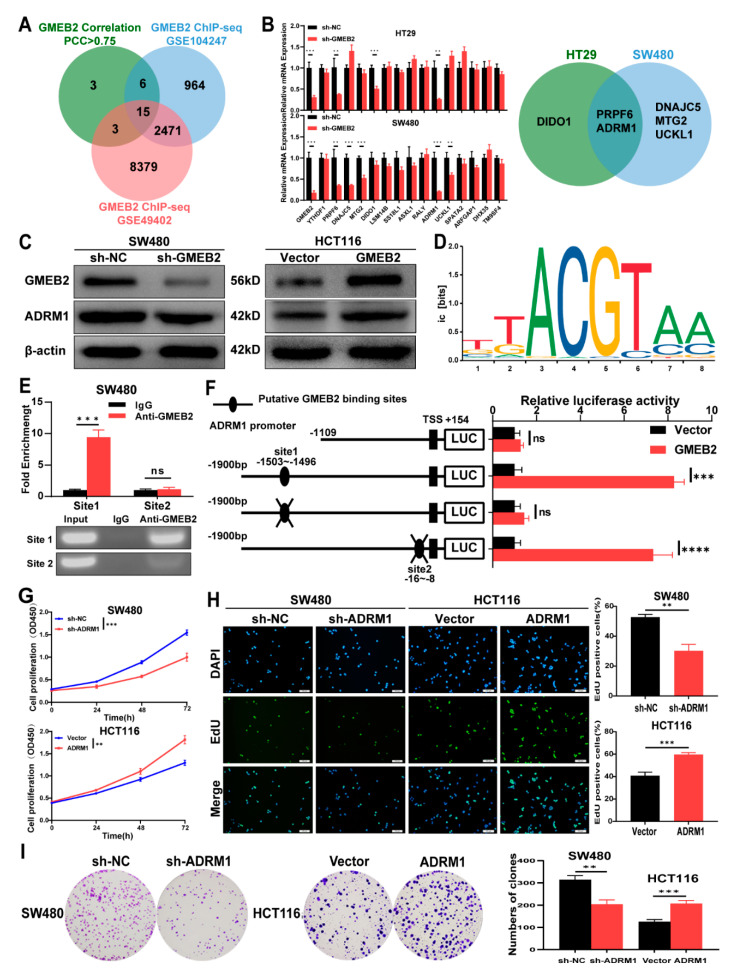
ADRM1, a direct transcriptional target of GMEB2, effectively facilitates CRC cell proliferation. (**A**) Venn diagram of GMEB2 ChIP-seq data (GSE104247, GSE49402) and GMEB2 correlation analyses in CRC (PCC > 0.75). (**B**) Genes expression after the GMEB2 knockdown in HT29 and SW480 cells was detected by qRT-PCR. The Venn diagram shows the correspondingly downregulated genes (Student’s *t*-test, ** *p* < 0.01, *** *p* < 0.001). (**C**) The relative protein level of ADRM1 in GMEB2 silenced or overexpressed CRC cells measured by western blotting. (**D**) The transcriptional factor GMEB2 binding motif from JASPAR database. (**E**) The ChIP-qPCR assay verified the direct binding of GMEB2 to the ADRM1 promoter in SW480 cells. (**F**) Dual-luciferase reporter assays on the wild-type, truncated and mutated ADRM1 promoter (TSS: transcription start site; ns *p* > 0.05, *** *p* < 0.001, **** *p* < 0.0001). (**G**) CCK-8 assays were used to detect the proliferation of SW480 and HCT116 cells after the knockdown or overexpression ADRM1 (two-way ANOVA, ** *p* < 0.01, *** *p* < 0.001). (**H**) EdU assays and the relative quantitative results of cell proliferation are shown in histogram form. (Student’s *t*-test, ** *p* < 0.01, *** *p* < 0.001). (**I**) Colony formation assays and the numbers of clones were counted (Student’s *t*-test, ** *p* < 0.01, *** *p* < 0.001). The results are presented as the mean ± S.D. of three independent experiments. *p* < 0.05. Scale bar, 100 µm.

To explore the downstream targets of the transcription factor GMEB2 in CRC, we analysed two GMEB2 chromatin immunoprecipitation sequencing (ChIP-seq) datasets, GSE104247 and GSE49402, in the GEO database (Tables S3 and S4). The GSE104247 dataset was obtained by ChIP-seq analysis in HepG2 liver cancer cells, while the GSE49402 dataset was obtained by ChIP-seq analysis in LoVo CRC cells. By performing correlation analyses in the GEPIA database, we obtained 27 genes whose Pearson correlation coefficient (PCC) with GMEB2 was >0.75 in CRC (Table S5). Finally, the intersection of the three gene datasets yielded 15 potential genes (Figure 3A). We detected the expression of these 15 genes by qRT-PCR in GMEB2-knockdown CRC cells and corresponding control cells and identified two significant genes that were both downregulated with GMEB2, namely PRPF6 and ADRM1 (Figure 3B). Previous studies have reported that ADRM1 is upregulated and promotes cell growth in CRC [27,28], while there have been few studies on the role of PRPF6 in tumour proliferation. Therefore, we chose ADRM1 as the downstream gene to further clarify the potential signalling pathways in which GMEB2 was involved. We verified the positive regulatory relationship between GMEB2 and ADRM1 at the protein level (Figure 3C). These findings suggest that GMEB2 positively modulated ADRM1 expression in CRC cells. To further explore the molecular mechanisms, we searched for a putative GMEB2 binding site in the promoter of ADRM1 (1900 bp, ADRM1-p; TSS, transcription start site 1) in the JASPAR (https://jaspar.genereg.net/, accessed on 16 September 2021) database and identified the two most conserved binding sites for GMEB2, located from −1503 bp to −1496 bp (site 1) and from −16 bp to −8 bp (site 2) (Figure 3D). ChIP-qPCR analysis was performed using an anti-GMEB2 antibody in CRC cells. The results showed that the site 1-binding signal was substantially increased, and a band of 90 bp containing site 1 was observed. Normal rabbit IgG was used as a negative control. However, neither enriched binding signals nor bands were evident in the immunoprecipitates for site 2 (Figure 3E). Dual-luciferase reporter assays showed that GMEB2 markedly transactivated ADRM1 promoter activity. Promoter truncation and site-directed mutagenesis indicated that site 1 of the ADRM1 promoter was the critical binding site for GMEB2 to induce ADRM1 transactivation, while no changes were observed for site 2 mutation (Figure 3F). These results suggest that GMEB2 bound to the consensus site between −1503 bp and −1496 bp on the ADRM1 promoter and activated the transcriptional activity of ADRM1.

Consistent with GMEB2, ADRM1 expression was analysed in the TCGA and GEO databases and found to be higher in CRC tissues than in normal tissues (Figure S2A,B). We similarly detected ADRM1 expression in the FHC cell line, as well as four CRC cell lines. The results showed that the mRNA and protein expression levels of ADRM1 were significantly higher in the CRC cell lines than in the FHC (Figure S2C,D). The expression levels of ADRM1 were positively correlated with GMEB2 in these CRC cell lines. However, previous studies on the function of ADRM1 in CRC cells were incomplete. To comprehensively verify the pro-proliferative effects of ADRM1 in CRC cells, we established ADRM1-silenced SW480 cells and ADRM1-overexpressing HCT116 cells. The ADRM1 silencing and overexpression effects were confirmed by qRT-PCR and western blotting (Figure S2E,F). Then, we performed CCK-8, EdU and colony formation assays to determine the role of ADRM1 in CRC cell growth. The results showed that ADRM1 facilitated the growth of CRC cells in vitro (Figure 3G–I and Figure S2G,H). Together, we concluded that ADRM1 was transactivated by the transcription factor GMEB2 and promoted CRC cell growth in vitro.

### 3.4. The GMEB2/ADRM1 Axis Promotes CRC Cell Growth In Vitro

**Figure 4:**
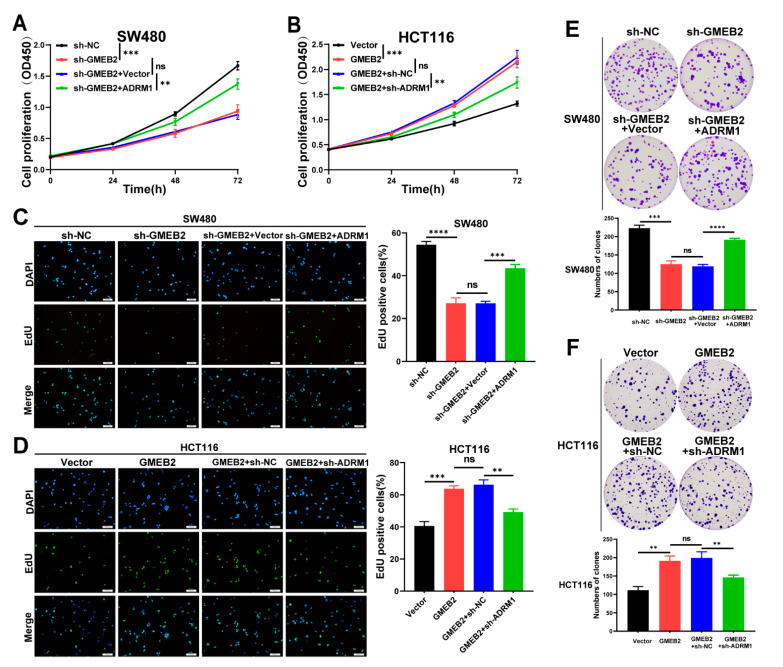
ADRM1 is essential for GMEB2-mediated CRC cell proliferation. (**A**,**C**,**E**) GMEB2-knockdown SW480 cells were transfected with the ADRM1-overexpressing plasmid for 48h, and the cell proliferation ability was quantified using the CCK-8, EdU and colony formation assays (Student’s *t*-test, ns *p* > 0.05, ** *p* < 0.01, *** *p* < 0.001, **** *p* < 0.0001). (**B**,**D**,**F**) GMEB2-overexpressing HCT116 cells were transfected with ADRM1 shRNA for 48h, and the cell proliferation ability was quantified using the CCK-8, EdU and colony formation assays (Student’s *t*-test, ns *p* > 0.05, ** *p* < 0.01, *** *p* < 0.001). The results are presented as the mean ± S.D. of three independent experiments. *p* < 0.05. Scale bar, 100 µm.

To investigate the role of ADRM1 in GMEB2-mediated CRC cell growth, we transfected ADRM1-overexpressing plasmids into GMEB2-knockdown SW480 cells (sh-GMEB2 + ADRM1), and empty vectors were transfected as a control (sh-GMEB2 + Vector). In addition, lentiviral ADRM1 shRNA was transfected into GMEB2-overexpressing HCT116 cells (GMEB2 + sh-ADRM1), and scrambled shRNA acted as a control (GMEB2 + sh-NC). We showed that ADRM1 overexpression restored the proliferative activity of GMEB2-knockdown SW480 cells by performing CCK-8, EdU and colony formation assays (Figure 4A,C,E). Similarly, ADRM1 knockdown inhibited HCT116 cell growth promoted by the overexpression of GMEB2 (Figure 4B,D,F). No significant difference was observed between the sh-GMEB2 and sh-GMEB2 + Vector groups or between the GMEB2 and GMEB2 + sh-NC groups. These results indicate that the empty vector and scrambled shRNA had no influence on cell growth. Collectively, these results prove that GMEB2 contributed to CRC cell proliferation by regulating ADRM1 in vitro.

### 3.5. The GMEB2/ADRM1 Axis Promotes Nuclear Translocation of NF-κB to Activate NF-κB Signalling

**Figure 5:**
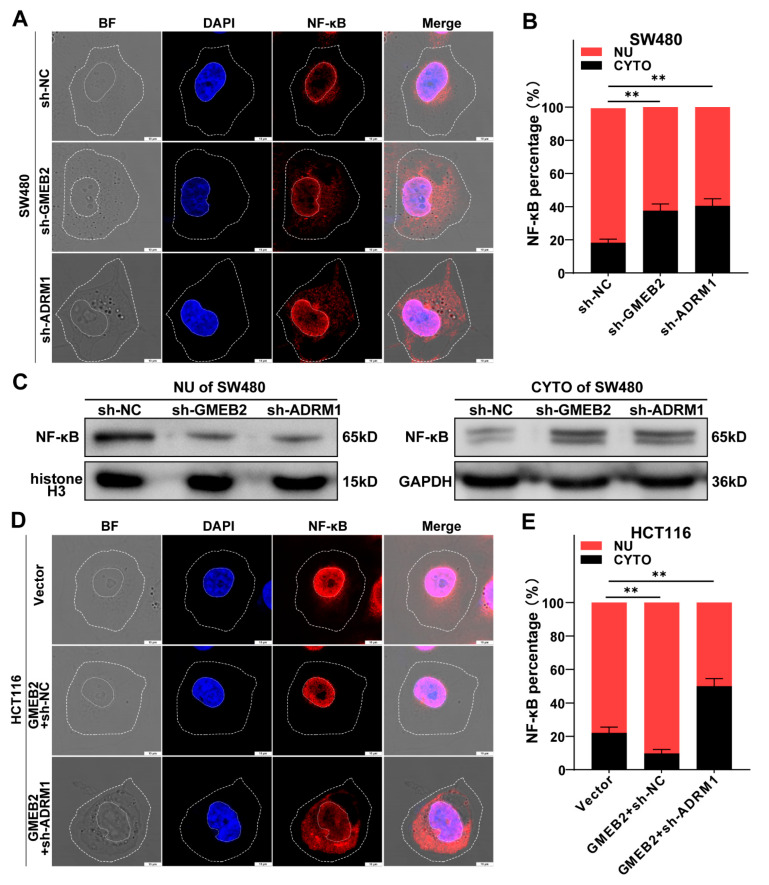
The GMEB2/ADRM1 axis can activate NF-κB signalling through increasing NF-κB nuclear translocation. (**A**) The confocal image showed NF-κB (p65) protein expression and localization in SW480 cells with GMEB2, ADRM1 or scrambled shRNA. (**B**) The proportion of fluorescence in the cytoplasm versus the nucleus was quantified for NF-κB (Student’s *t*-test, ** *p* < 0.01). (**C**) Western blotting showed NF-κB localization in SW480 cells with GMEB2, ADRM1 or scrambled shRNA, histone-H3 (a nuclear marker) and GAPDH (a cytoplasmic marker) as controls. (**D**) The confocal image showed NF-κB localization in GMEB2-overexpressing HCT116 cells transfected with the ADRM1-knockdown plasmid. (**E**) The proportion of fluorescence in the cytoplasm versus the nucleus was quantified for NF-κB (Student’s *t*-test, ** *p* < 0.01). Scale bar, 10 µm.

A previous study reported that ADRM1 promoted the proteasomal degradation of IκBα to regulate the activity of transcription factor NF-κB (p65) [40]. IκBα degradation and subsequent nuclear translocation of NF-κB are crucial for activating the canonical NF-κB pathway [41]. Thus, we intended to confirm the effect of the GMEB2/ADRM1 axis on NF-κB signalling in CRC cells. In SW480 cells, we silenced GMEB2 and ADRM1 and observed the effects on NF-κB translocation into the nucleus compared with negative control cells. We visualized the intracellular distribution of NF-κB by immunofluorescence. The majority of the NF-κB protein was still located in the nucleus. However, the knockdown of GMEB2 or ADRM1 observably increased the amount of NF-κB in the cytoplasm (Figure 5A). The nucleocytoplasmic distribution of NF-κB was quantified by ImageJ software, and the results showed that the cytosolic fraction was significantly higher in GMEB2- or ADRM1-silenced cells (Figure 5B). After extracting cytosolic and nuclear proteins, we observed a similar trend in the western blotting results (Figure 5C). Moreover, ADRM1 knockdown completely reversed the GMEB2 overexpression-mediated promotion of NF-κB nuclear translocation (Figure 5D,E). Collectively, these observations suggest that the GMEB2/ADRM1 axis enhanced NF-κB nuclear translocation and thus activated NF-κB signalling.

### 3.6. GMEB2 mRNA Is Stabilized by YTHDF1 in an m^6^A-Dependent Manner

**Figure 6:**
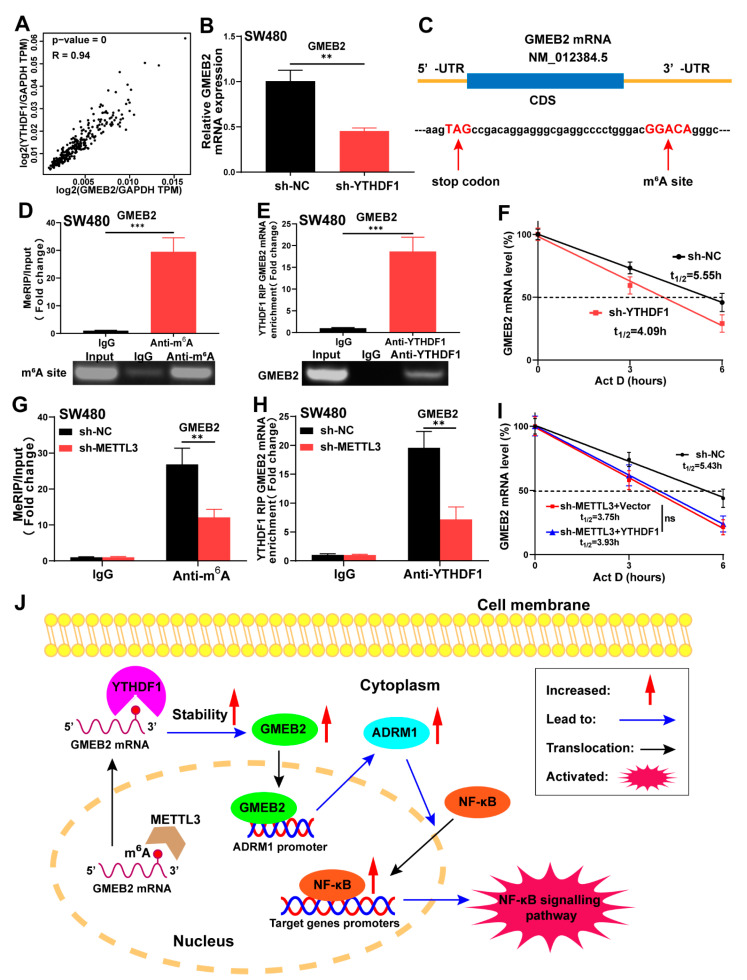
YTHDF1 can stabilize GMEB2 mRNA through recognizing METTL3-mediated m^6^A modification. (**A**) Correlation of mRNA expression between GMEB2 and YTHDF1 in CRC from GEPIA database. (**B**) GMEB2 mRNA expression in the YTHDF1 knockdown SW480 cells (Student’s *t*-test, ** *p* < 0.01). (**C**) The m^6^A site in the 3′-UTR of GMEB2 mRNA (near stop codon). (**D**) MeRIP-qPCR indicated the relative GMEB2 mRNA enrichment precipitated by m^6^A antibody (Student’s *t*-test, *** *p* < 0.001). (**E**) RIP-qPCR indicated the direct binding within YTHDF1 and GMEB2 mRNA on the m^6^A site (Student’s *t*-test, *** *p* < 0.001). (**F**) The RNA decay rate followed by the qRT-PCR assay demonstrated the changes in GMEB2 mRNA stability upon the YTHDF1 knockdown. Data were detected at the indicated timepoint with actinomycin D (Act D, 5 μg/mL) treatment (Student’s *t*-test, *p* < 0.01). (**G**) MeRIP-qPCR indicated the relative GMEB2 mRNA enrichment after METTL3 knockdown (Student’s *t*-test, ** *p* < 0.01). (**H**) RIP-qPCR showed the change of YTHDF1′s ability to bind GMEB2 mRNA after METTL3 knockdown (Student’s *t*-test, ** *p* < 0.01). (**I**) The RNA stability assay showed GMEB2 mRNA half-lives in METTL3-knockdown SW480 cells with the YTHDF1 overexpressed (Student’s *t*-test, ns *p* > 0.05). (**J**) A schematic diagram illustrating the role of GMEB2 in CRC. GMEB2 transactivates ADRM1 transcription to promote nuclear translocation of NF-κB, and its mRNA is stabilized by YTHDF1 in an m^6^A-dependent manner.

As mentioned above, we performed correlation analysis in the GEPIA database and showed that YTHDF1 was highly correlated with GMEB2 in CRC (Figure 6A). We also found that YTHDF1 was highly expressed in CRC from TCGA and GEO databases analyses (Figure S3A,B). However, GMEB2 knockdown did not affect the expression of YTHDF1 in CRC cells (Figure 3B). We established YTHDF1-silenced SW480 cells with lentiviral vectors carrying YTHDF1-specific small hairpin RNA (sh-YTHDF1). Negative control cells were transfected with lentiviral vectors carrying scrambled shRNA (sh-NC). The knockdown of YTHDF1 was validated by qRT-PCR and western blotting (Figure S3C,D). We demonstrated that YTHDF1 silencing led to the decreased expression of GMEB2 (Figure 6B, S3D). Considering that YTHDF1 is the most powerful m^6^A reader protein and was found to potentially bind with GMEB2 mRNA by RNA immunoprecipitation-sequencing (RIP-seq) in a previous study [15], we speculated that YTHDF1 may function as an upstream regulator of GMEB2 by mediating m^6^A modification. First, the m^6^A modification sites on GMEB2 mRNA were predicted by the sequence-based RNA adenosine methylation site predictor (SRAMP) website (Figure 6C and Figure S3E). Next, the methylated RIP (MeRIP)-qPCR results verified the m^6^A site in the 3′-UTR of GMEB2 mRNA near the stop codon, and a band of 147 bp was observed, containing the m^6^A site (Figure 6D). Similarly, RIP-qPCR demonstrated direct binding of YTHDF1 to the m^6^A site of GMEB2 mRNA in SW480 cells (Figure 6E). The RNA stability assay showed that the downregulation of YTHDF1 reduced the stability of GMEB2 mRNA, as its mRNA half-life was significantly shortened (Figure 6F). The GMEB2 m^6^A motif is GGACA, in which METTL3 can catalyse the methylation of adenylate. We constructed METTL3-silenced SW480 cells (Figure S3F,G). The knockdown of METTL3 decreased the m^6^A modification level of GMEB2 mRNA (Figure 6G) and impaired YTHDF1′s ability to bind to GMEB2 mRNA (Figure 6H). Finally, we overexpressed YTHDF1 in METTL3-silenced SW480 cells and found that the stability of GMEB2 mRNA did not change, which indicated that YTHDF1 mediated GMEB2 mRNA stability in an m^6^A-dependent manner (Figure 6I). In addition, there is no m^6^A modification site on ADRM1 mRNA, as confirmed by previous MeRIP-seq data [15] and our MeRIP-qPCR results (Figure S3H). This finding indicates that there is no binding site for YTHDF1, which is consistent with the YTHDF1 RIP-seq results [15]. In summary, these findings demonstrate that GMEB2 was positively regulated by YTHDF1 in an m^6^A-dependent manner and served as a transcription factor to activate the ADRM1/NF-κB signalling pathway, which contributed to CRC growth (Figure 6J).

## 4. Discussion

In this study, we confirmed, for the first time, that GMEB2 expression was highly increased in both CRC tissues and cell lines. Next, we demonstrated that GMEB2 significantly promoted the growth of CRC in vitro and in vivo. Mechanistically, we revealed that GMEB2 acted as a transcription factor to increase the transcription of ADRM1. Moreover, the GMEB2/ADRM1 axis activated NF-κB signalling by promoting the nuclear translocation of NF-κB in CRC. Finally, we clarified the mechanism of GMEB2 upregulation in CRC. The GMEB2 mRNA was stabilized by YTHDF1 in an m^6^A-dependent manner.

To date, emerging evidence indicates that the dysregulation of transcription factors exerts important tumour-promoting effects in various types of cancer through diverse mechanisms. For example, the hyperactivation of signal transducer and activator of transcription 3 (STAT3) contributes to the malignant progression of CRC by mediating key inflammatory mechanisms [42]. Approaches of targeting transcription factor activity highlight the clinical potential of cancer treatments [43], as an oncogenic transcription factor could aberrantly activate a number of oncogenes in signalling pathways. GMEB2 possesses intrinsic transactivation activities [5], which may play significant roles in tumours. We found that GMEB2 expression was upregulated in CRC by analysing TCGA and GEO data. However, there is no detailed study in cancer that has identified the role of GMEB2 as a transcription factor. First, we confirmed that GMEB2 expression was higher in 12 human CRC tissues than in the matched adjacent non-tumour tissues. Next, we determined the biological function of GMEB2 in CRC. GMEB2 was proven to promote CRC cell growth in vitro and in vivo for the first time. These results suggest that GMEB2 may play an important role in promoting the development and progression of CRC and may be a possible therapeutic target for CRC treatment.

Aberrant RNA m^6^A modifications are frequently exhibited in tumours and are involved in tumour proliferation, metastasis, angiogenesis and immunity [44]. M^6^A plays a dual role during the pathogenesis of cancer depending on its regulators and the distinct pathways involved [45,46]. Interestingly, the m^6^A reader YTHDF1 plays an oncogenic role in cancer. In particular, high expression of YTHDF1 is associated with poor overall survival and malignant tumour behaviours in CRC [47]. In addition to its primary function in promoting protein translation [23], YTHDF1 was recently reported to promote the stability of mRNAs via m^6^A modifications in multiple cancers, such as gastric cancer (GC) [48], hepatocellular carcinoma (HCC) [49] and cervical cancer [50]. Here, we found an m^6^A modification site in GMEB2 mRNA that was recognized by YTHDF1 to stabilize its mRNA. When the m^6^A writer METTL3 was knocked down, YTHDF1 could no longer regulate GMEB2 mRNA stability, which means that YTHDF1 exerted its effect on GMEB2 in an m^6^A-dependent manner. Our results elucidated the upregulation mechanism of GMEB2 and provide more detailed and reliable evidence to prove the significant role of GMEB2 in CRC.

Hyperactivation of the NF-κB signalling pathway is a characteristic feature of CRC, regulating the proliferation, apoptosis, metastasis and inflammation of CRC [51]. Degradation of IκBα is key for inducing the translocation of the transcription factor NF-κB into the nucleus, activating the expression of numerous oncogenes in CRC [41]. Previous studies have shown that the ADRM1 protein regulates the ubiquitination and degradation of IκBα to activate the transcription factor NF-κB [40]. Moreover, by specifically causing ADRM1 to lose its function, the agent RA190 blocks the nuclear translocation of NF-κB and the consequent activation of the NF-κB signalling pathway in HCC cells [36]. However, the underlying regulatory mechanism of the ADRM1/NF-κB pathway has not been investigated. Here, we identified the direct binding of GMEB2 to the promoter of the ADRM1 gene by ChIP-qPCR and dual-luciferase reporter assays. Furthermore, we verified that the GMEB2-ADRM1 axis induced nuclear translocation of NF-κB, thus activating NF-κB signalling in CRC cells. These results indicate that GMEB2 may promote CRC growth by enhancing the constitutive expression of proliferation-associated genes in the NF-κB pathway, such as cyclin D1, cyclin E and Myc [52]. It may be a similar mechanism to the anti-apoptotic effect of GMEB2 in CRC. Overall, our exploration of the regulatory mechanism of the effect of GMEB2 on the ADRM1/NF-κB pathway provides new insights into the tumorigenesis and treatment of CRC.

## 5. Conclusions

In summary, our findings demonstrate that GMEB2 was highly expressed in CRC and significantly promoted the growth of CRC in vitro and in vivo. Mechanistically, GMEB2 acted as a transcription factor to transactivate the ADRM1/NF-κB pathway and was upregulated by YTHDF1 by enhancing its mRNA stability. Taken together, our results suggest the critical role and regulatory mechanism of GMEB2 in CRC and that GMEB2 is a new potential therapeutic target for CRC treatment in the future.

## Data Availability

The data presented in this study are available from the corresponding author upon reasonable request.

## Supplementary Materials

The following supporting information can be downloaded at: https://www.mdpi.com/article/10.3390/cancers14246046/s1, Figure S1: Knockdown of GMEB2 inhibits CRC cell proliferation; Figure S2: The mRNA and protein expression of ADRM1 in CRC cells; Figure S3: YTHDF1 can positively regulate GMEB2 expression; Table S1: Primers and shRNA sequences; Table S2: Primary and secondary antibodies; Table S3: GMEB2 ChIP-seq dataset in GSE104247; Table S4: GMEB2 ChIP-seq dataset in GSE49402; Table S5: The Pearson correlation analysis results for GMEB2 with PCC > 0.75.
