# Supplementary material for: GMEB2 Promotes the Growth of Colorectal Cancer by Activating ADRM1 Transcription and NF-κB Signalling and Is Positively Regulated by the m6A Reader YTHDF1
Source: Cancers (Basel). 2022 Dec 8;14(24):6046. doi: 10.3390/cancers14246046 (PMC9776391; doi:10.3390/cancers14246046)
Supplement: Supplementary file 1 [file cancers-14-06046-s001.zip › Supplementary Figure S1-S3.pdf]

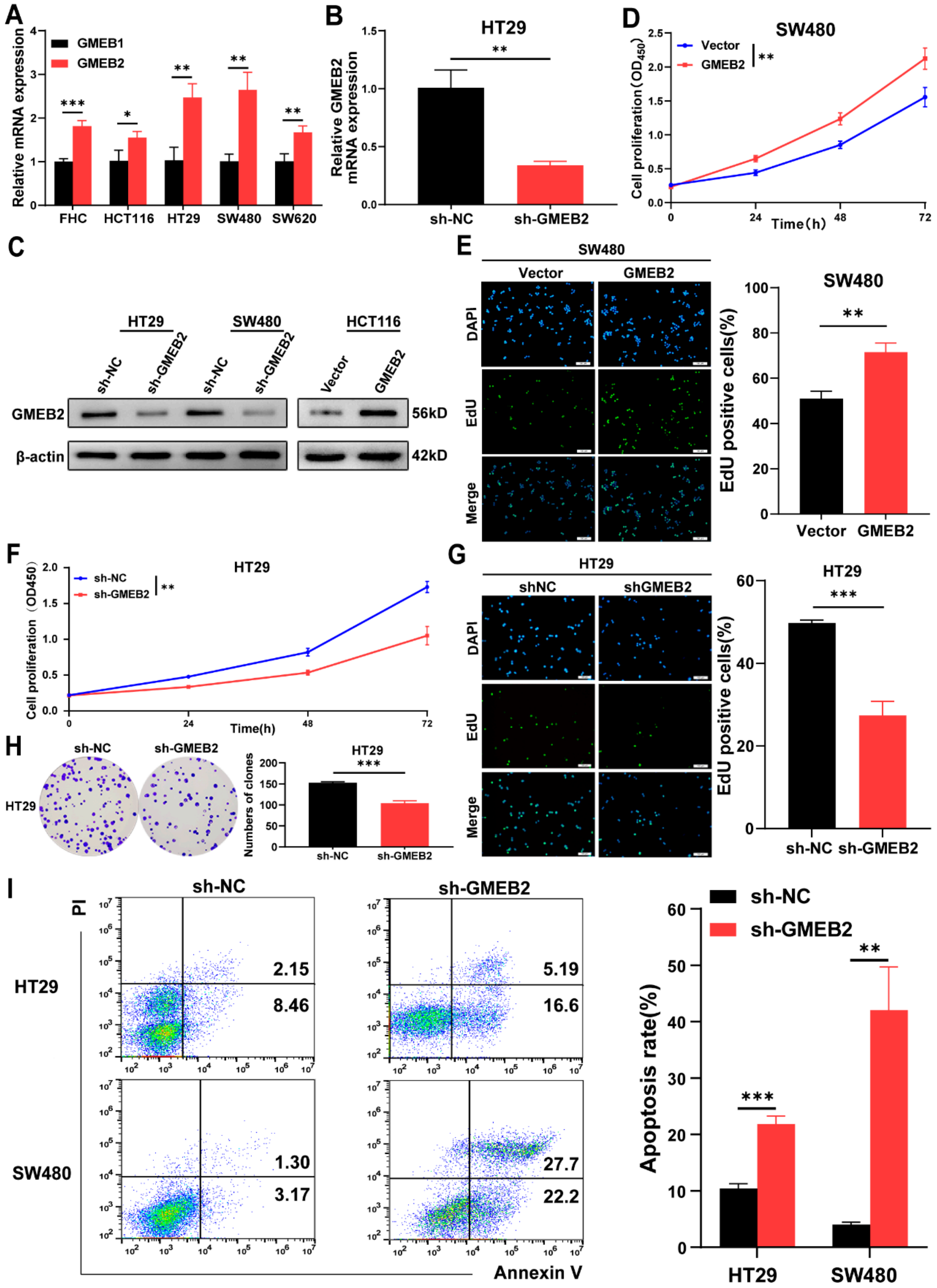

Figure S1. Knockdown of GMEB2 inhibits CRC cell proliferation. (**A**) GMEB1 and GMEB2 relative mRNA levels in FHC and CRC cell lines (HCT116, HT29, SW480 and SW620) (Student's t-test, *p < 0.05, **p < 0.01, ***p < 0.001). (**B**) Silencing efficiency of GMEB2 in HT29 cells measured by qRT-PCR (Student's t-test, **p < 0.01). (**C**) Silencing or overexpression efficiencies of GMEB2 in SW480, HT29 and HCT116 cells measured by western blotting. (**D, F**) CCK-8 assay was used to detect the proliferation of SW480 and HT29 cells after overexpression or knockdown

GMEB2 (two-way ANOVA, **p < 0.01). (**E, G**) EdU assay and the relative quantitative result of cell proliferation are shown in histogram form (Student's t-test, ***p < 0.001). (**H**) The colony formation assay and numbers of clones were counted (Student's t-test, ***p < 0.001). The results are presented as the mean ± S.D. of three independent experiments. p < 0.05. (**I**) Annexin V-PI staining followed by cytometry analysis was performed to evaluate the cell apoptosis of shGMEB2 HT29 and SW480 cells and their corresponding NC cells. **p < 0.01, ***p < 0.001. Scale bar, 100 μm.

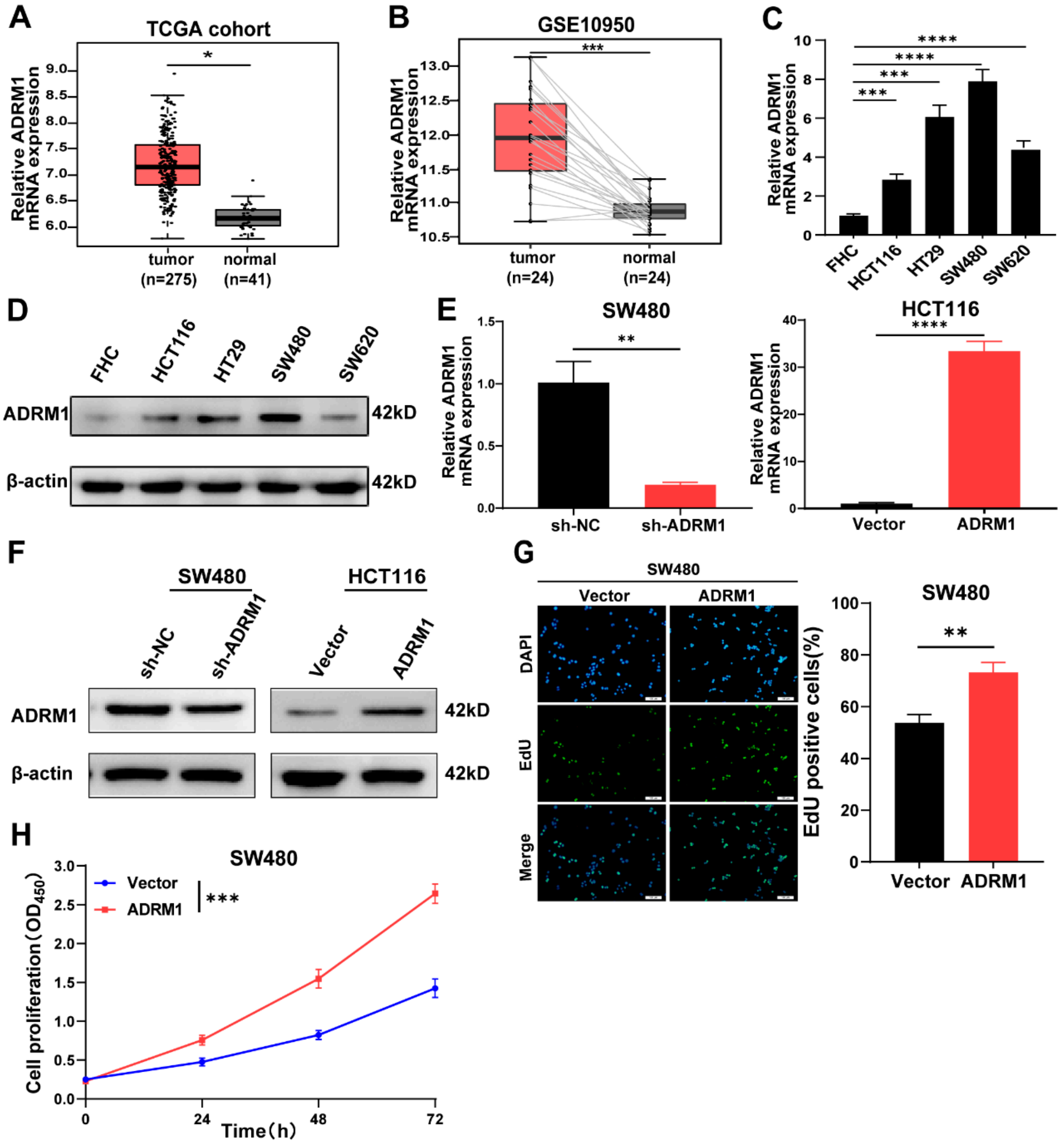

Figure S2. The mRNA and protein expression of ADRM1 in CRC cells. (**A**) ADRM1 mRNA level in the TCGA database (Student's t-test, *p < 0.05). (**B**) ADRM1 mRNA level in GSE10950 of the GEO database (paired Student's t-test, ***p <0.001). (**C**) ADRM1 mRNA levels in FHC and CRC cell lines (HCT116, HT29, SW480 and SW620) (Student's t-test, ***p < 0.001, ****p < 0.0001). (**D**) ADRM1 protein levels in the FHC and CRC cell lines (HCT116, HT29, SW480 and SW620) measured by western blotting. (**E**) Silencing or overexpression efficiencies of ADRM1 in SW480 and HCT116 cells measured by qRT-PCR (Student's t-test, **p < 0.01, ***p < 0.001). (**F**) Silencing or overexpression efficiencies of ADRM1 in SW480 and HCT116 cells measured by western

blotting. (**G**) EdU assay and the relative quantitative result of cell proliferation were shown in histogram form after overexpressing ADRM1 in SW480 cells (Student's t-test, **p < 0.01). (**H**) A CCK-8 assay was used to detect the proliferation of ADRM1-overexpressed SW480 cells (two-way ANOVA, ***p < 0.001).

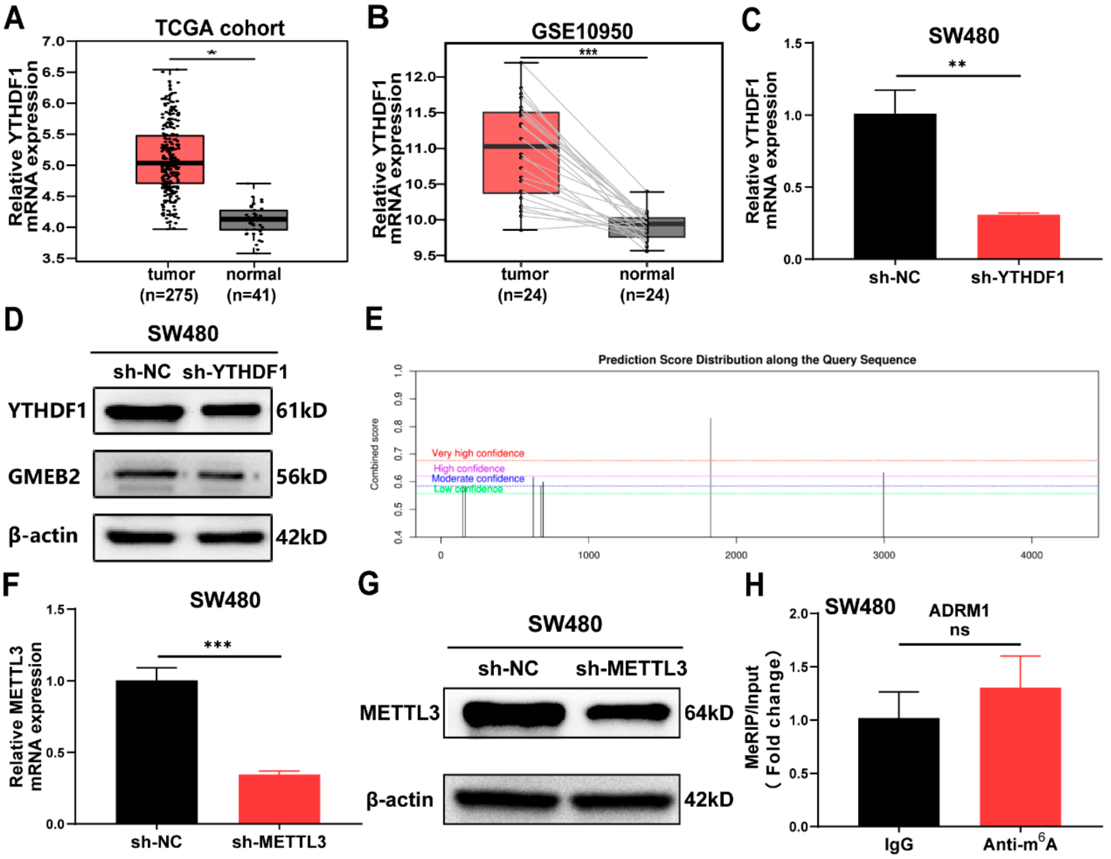

Figure S3. YTHDF1 can positively regulate GMEB2 expression. (**A**) YTHDF1 mRNA level in the TCGA database (Student's t-test, *p < 0.05). (**B**) YTHDF1 mRNA level in GSE10950 of the GEO database (paired Student's t-test, ***p <0.001). (**C**) Silencing efficiency of YTHDF1 in SW480 cells measured by qRT-PCR (Student's t-test, **p < 0.01). (**D**) Silencing efficiency of YTHDF1 and subsequent GMEB2 expression in SW480 cells measured by western blotting. (**E**) The potential m^6A modification sites on GMEB2 mRNA were predicted by SRAMP. (**F**) Silencing efficiency of METTL3 in SW480 cells measured by qRT-PCR (Student's t-test, ***p < 0.001). (**G**) Silencing efficiency of METTL3 in SW480 cells measured by western blotting. (**H**) MeRIP-qPCR showed no ADRM1 mRNA enrichment precipitated by the m^6A antibody (Student's t-test, ns p > 0.05).
