# Supplementary material for: GMEB2 Promotes the Growth of Colorectal Cancer by Activating ADRM1 Transcription and NF-κB Signalling and Is Positively Regulated by the m6A Reader YTHDF1
Source: Cancers (Basel). 2022 Dec 8;14(24):6046. doi: 10.3390/cancers14246046 (PMC9776391; doi:10.3390/cancers14246046)
Supplement: Supplementary file 1 [file cancers-14-06046-s001.zip › Supplementary Table S1.pdf]

Supplementary Table S1. Primers and shRNA sequences

Application	Target gene	Forward sequence (5' to 3')	Reverse sequence (5' to 3')
qRT-PCR primers	human GMEB2	GGCGACCTGACGGAAGATAAC	TCGTCGTACTGAACACATTTCAC
	human ADRM1	TTCAGCAGACGGACGACTC	CACCCGCTTGAACTCACAG
	human YTHDF1	ATACCTCACCACCTACGGACA	GTGCTGATAGATGTTGTTCCCC
	human METTL3	TTGTCTCCAACCTTCCGTAGT	CCAGATCAGAGAGGTGGTGTAG
	human GAPDH	ACCTGACCTGCCGTCTAGAA	TCCACCACCCTGTTGCTGTA
	human PRPF6	GAGGATGCTGACAGTTGTGTAG	CCATGGTTCTTCTCGAAGTACG
	human DNAJC5	GGGAGTCATTGTACCACGTCC	CGTGCGCGTTGTTGATCTC
	human MTG2	TTTGTGGACTATCGGAGAGTGC	CGACGACAGGGACTTGACTT
	human DIDO1	AAAGGCGACCCGAGCAATG	GCTTGGCGATAGTGGTCCTTC
	human LSM14B	GAAGACCGTCCCACAGATAGG	GCTGAGCTTTCGGAGGTTCA
	human SS18L1	CAAGGCACCATCGGCAACTA	CTGCTGCATCATGGAGACTG
	human ASXL1	CGCGCCTGGTATTAGAAAACT	GCATCCTTCTTGAGCGTGAAAAG
	human RALY	GCACAAGGGCTATGCCTTTG	CTTAGGCTCTCCAGCCATGTT
	human UCKL1	AATGAACACGGCACGCAATC	GCTCAGTCAGCACCTTGTAGAA
	human SPATA2	CCTCTACCCGTGGAAGAAGGA	TCTGCTCACACTCGACTTTGG
	human ARFGAP1	CTTTAGGGATAAGGTGGTCGCT	AGGTCATCATTCAGCCAGTCT
	human DHX35	GCTGCTGTTACAGTTGCAGG	CTGGTCGGTGCAGTCATCAAA
	human TM9SF4	GATTGGTTGCCGTGGTCTTTA	TTCTACGGGATCGTTCTGGTG

ChIP-qPCR primers	ADRM1 promoter 1	CTGCCAGCATCAACACCAACG	ACAGCTGTGTGCTTACGTGCT
	ADRM1 promoter 2	CACAGCAACAATAGGGGCCG	GGCTCTAACCTGCCAACGAT
MeRIP and RIP –qPCR primers	GMEB2 mRNA	AGCGGAAGTAGCCGACA	GCGATTCCTCTCTTTGGTTCTG
shRNA	human GMEB2	GTTCAGTACGACGAGCATGTA	
	human ADRM1	AGTCAACGAGTATCTGAACAA	
	human YTHDF1	ATCGGTCTAAAGTGCTAATTT	
	human METTL3	CTGCAAGTATGTTCACTATGA	
