# Supplementary material for: GMEB2 Promotes the Growth of Colorectal Cancer by Activating ADRM1 Transcription and NF-κB Signalling and Is Positively Regulated by the m6A Reader YTHDF1
Source: Cancers (Basel). 2022 Dec 8;14(24):6046. doi: 10.3390/cancers14246046 (PMC9776391; doi:10.3390/cancers14246046)
Supplement: Supplementary file 1 [file cancers-14-06046-s001.zip › Supplementary Table S2.pdf]

Supplementary Table S2. Primary and secondary antibodies

Antibody	Application	Host	Vendor
anti-GMEB2	WB, IHC	Rabbit	Bioss, #bs-13456R
anti-GMEB2	ChIP	Mouse	Santa Cruz, #sc-81093
anti-ADRM1	WB	Rabbit	Zen Bio, #R23396
anti-YTHDF1	WB, RIP	Rabbit	Proteintech, #17479-1-AP
anti-METTL3	WB	Rabbit	Proteintech, #15073-1-AP
anti-NF-κB	WB, IF	Mouse	Zen Bio, #250060
anti-β-actin	WB	Rabbit	Proteintech, # 20536-1-AP
anti-GAPDH	WB	Rabbit	Proteintech, #10494-1-AP
anti-Histone-H3	WB	Rabbit	Proteintech, #17168-1-AP
HRP anti-Rabbit IgG	WB	Goat	ABclonal, #AS029
HRP anti-Mouse IgG	WB	Goat	ABclonal, #AS003
Dylight 594 anti-Mouse IgG	IF	Goat	Immunoway, #RS23410
